# Exceeding the guideline-recommended maximum daily dose of opioids for long-term treatment of non-cancer pain in Germany – a large retrospective observational study

**DOI:** 10.1186/s12889-024-20141-4

**Published:** 2024-09-27

**Authors:** Nils Frederik Schrader, Anja Niemann, Milena Weitzel, Christian Speckemeier, Carina Abels, Nikola Blase, Godwin Denk Giebel, Cordula Riederer, Joachim Nadstawek, Wolfgang Straßmeir, Jürgen Wasem, Silke Neusser

**Affiliations:** 1 Institute for Healthcare Management and Research, University of Duisburg-Essen, Thea-Leymann-Str. 9, Essen, Nordrhein-Westfalen 45127 Germany; 2 DAK-Gesundheit, Hamburg, Germany; 3 Association of German Doctors and Psychotherapists practicing in Pain Medicine and Palliative Care – BVSD e.V, Berlin, Germany

**Keywords:** Opioid analgesics, Non-cancer pain, Long-term opioid treatment, Guideline, Maximum daily doses, Social insurance data

## Abstract

**Background:**

High-dose long-term opioid therapy (LTOT) has been associated with increased mortality and hospitalizations. Therefore, the evidence-based German guideline on LTOT for chronic non-cancer pain (CNCP) recommends to only exceed the maximum daily dose (MDD) of opioids in exceptional cases. This study aimed to determine the portion of LTOT patients who exceeded the guideline-recommended MDD and identify predictors of exceeding in administrative claims data.

**Methods:**

The retrospective observational analysis of opioid prescriptions in patients receiving LTOT for CNCP was based on administrative claims by a large German statutory health insurance company. Patients with at least two quarters of opioid prescriptions between January 2018 and June 2019 were included and followed up for two years. Predictors were identified by logistic regression. In addition, the number of patients still in opioid therapy and the extent of exceeded MDDs were analyzed over time.

**Results:**

The sample consisted of 113,475 patients. Overall, 10.5% of the patients exceeded the guideline-recommended MDD averaged over the observation period. Strong predictors for exceeding the MDD were receiving opioid prescriptions from > 7 physicians (OR = 7.66, *p* < .001), receiving predominantly strong opioids (OR = 6.79, *p* < .001) and receiving opioids for at least one year prior to inclusion (OR = 5.35, *p* < .001). Within the non-exceeding group, 28.1% discontinued opioid therapy. In contrast, 9.9% of patients in the exceeding group discontinued opioid therapy, whereas the vast majority remained on treatment until the end of the observation period. Furthermore, a slight increase in prescribed doses was observed over time.

**Conclusions:**

The results indicate that a moderate proportion of patients exceeded the guideline-recommended MDD. However, certain patient groups were more likely to receive high doses. This applied in particular to those who were already on treatment at the time of inclusion and continued to receive opioids until the end of the observation period. Further research should examine whether the continuous opioid therapy among the patients with exceeding the guideline-recommended MDD might be related to specific indications, a lack of therapeutic options or avoidance of withdrawal.

**Trial registration:**

German Clinical Trials Register (drks.de/search/en). Identifier: DRKS00024854. Registered 28 April 2021.

**Supplementary Information:**

The online version contains supplementary material available at 10.1186/s12889-024-20141-4.

## Background

Chronic non-cancer pain (CNCP) is a common health burden and often treated with long-term opioid therapy (LTOT), which can be defined as opioid therapy for at least three months [1, 2]. According to various studies, the number of opioid patients with CNCP [3–5] but also the number of prescribed defined daily doses (DDD) [5, 6] have been increasing over the last two decades in Germany. Although there is no evidence for an opioid crisis of epidemic proportions [7, 8], this development demands careful attention. Due to its various potential side effects such as misuse, opioid prescriptions are strictly regulated by law in Germany [9]. Additionally, an evidence-based guideline addresses LTOT for CNCP in Germany to guide clinicians through interdisciplinary therapy concepts and increase its safety [1].

The guideline recommends to only exceed the maximum daily dose (MDD) of 120 oral morphine milligram equivalents (oMME) in exceptional cases [1]. Doses > 120 oMME/day are associated with a higher risk of car accidents [10], hospitalizations [2, 11] and increased mortality [12]. Non-guideline-compliant dosing is also associated with higher healthcare costs [2], whereas dose escalation can lead to opioid-induced tolerance and hyperalgesia [13]. A 2016 meta-analysis of chronic low back pain reported only modest pain relief at high doses for patients on short-term opioid therapy [14]. In addition, a recent Cochrane review found a lack of evidence for the efficacy of opioid therapy at doses above 200 oMME/day [15].

The only studies that analyzed high-dose LTOT in Germany used claims data from 2012 and 2014, and focused on pain as medical indications, comorbidities, comedication and indicators of substance abuse [2, 16, 17]. To our knowledge, no German study has examined the relationship between doses and the characteristics of prescribed opioid analgesics (OAs) yet. Furthermore, analyses of daily doses over the course of therapy are lacking for Germany. In addition, the studies identified by us, defined high-dose therapy based on fixed thresholds [2, 16], e.g. persons receiving more than 120 oMME/day [2]. We modified this approach slightly and reverted to opioid-specific thresholds instead, as the recommended MDD of some opioids is below 120 oMME, e.g. in tilidine-naloxone combinations or tramadol [18, 19]. The German guideline itself supplements its recommendations with a practical tool indicating that some OAs have MDDs below 120 oMME [20].

Therefore, the objective of this study was to examine whether and to what extent the guideline-recommended MDDs of OAs are exceeded among patients receiving LTOT for CNCP insured by a large German statutory health insurance (SHI) company. Given the availability of a two-year observation period, it was possible to determine the number of patients with continued opioid therapy and the extent to which guideline-recommended MDDs were exceeded among the cohort over time. Further, we aimed to identify predictors of exceeding the guideline-recommended MDD using a logistic regression model. In addition to factors previously associated with high-dose LTOT (e.g., age, sex, comedication), we included characteristics that have not been extensively studied to date, such as prescribed opioid agents, the history of OA prescriptions, and prescriptions from pain therapists. To provide a more comprehensive picture of opioid prescribing, doses, combinations of prescribed opioids, route of administration (RoA) and prescribing specialist groups were analyzed descriptively.

## Methods

### Data set and sampling description

The analysis was based on administrative claims data from the German SHI company *DAK-Gesundheit*, which insured approximately 5.7 M persons in 2018 [21]. The data covered pharmaceutical prescriptions and health care utilization from January 2018 to March 2021. Patients were included within the period from January 2018 to June 2019 (selection period) if they were > 17 years old, received at least one prescription of OA per quarter in two successive quarters and had no diagnoses of malignancies (ICD-10-GM (International Statistical Classification of Diseases and Related Health Problems, 10th revision, German Modification) C00-C97) or indicators of palliative care (ICD-10-GM Z51.5; EBM (Uniform Assessment Standard) 01425–01426, 03370–03373, 04370–04373, 37,300–37320 from outpatient doctor’s fee schedule; German-DRG ZE60, ZE145, ZE2020-133, ZE2020-134 for inpatient billing; OPS (German procedural classification) 8–982, 8–98e, 8–98 h, 1–265.b, 1–773, 1–774) within that two quarters. The observation period for each patient started on the date of the first prescription that led to enrollment. After inclusion, patients were followed up for two years or until death. Table 1 shows the ATC codes of OAs used for patient selection. Methadone and levomethadone were excluded because they are typically utilized for the treatment of heroin addiction in Germany [6]. The prescription data covered outpatient prescriptions only. A detailed description of the selection process can be found in Schrader & Niemann et al. [22]. To obtain further information on the prescribed DDD per package and the RoA, the claims data were merged with complementary drug-specific data from a German pharmaceutical database [23].

**Table 1:** Opioid analgesics used for patient selection (Source: [24])

Opioid analgesics	ATC
Morphine	N02AA01
Hydromorphone	N02AA03
Oxycodone	N02AA05
Oxycodone/naloxone	N02AA55
Fentanyl	N02AB03
Buprenorphine	N02AE01
Tilidine/naloxone	N02AX51
Tramadol	N02AX02
Tramadol/paracetamol	N02AJ13
Tapentadol	N02AX06

### Opioid dose conversion and calculation

The dose conversion was based on the data from Table 2 to obtain a prescribed oMME-dose per day for each patient. We calculated the ratio of the prescribed daily dose to the MDD to determine the number of prescribed MDDs for each patient and to examine whether a patient exceeded the guideline-recommended MDD.

**Table 2:** Conversion factors and Maximum Daily Doses used for dose conversion and calculation of prescribed doses

	**Conversion factors**		**Guideline-recommended maximum daily dose**		
**Opioid**	**(1)** **DDD to mg**^**a**^	**(2)** **mg to oMME**^**b**^	**(3)** **mg**^**b**^	**(4)** **oMME**^**b, i**^	**(5)** **Correction factor**^**j**^
**Morphine**					
Oral	100	1	120	120	-
Parenteral	30	3^c^	40	120	-
Rectal	30	1^d^	120	120	-
**Hydromorphone**					
Oral	20	7.5	16	120	-
Parenteral	4	15^c^	8	120	-
**Oxycodone**					
Oral	75	2	60	120	-
Parenteral	30	3^c^	40	120	-
**Oxycodone/naloxone**					
Oral	75	2	40	80	1.5
**Fentanyl**					
Transdermal	1.2	100	1.2	120	-
Sublingual/buccal	0.6	130^e^	0.92	120	-
Nasal	0.6	160^e^	0.75	120	-
**Buprenorphine**					
Transdermal	1.2	75	1.6	120	-
Sublingual/buccal	1.2	100^f^	1.2	120	-
Parenteral	1.2	100^g^	1.2	120	-
**Tilidine/naloxone**					
Oral	200	0.1	600	60	2
**Tramadol**					
Oral	300	0.1	400	40	3
Parenteral	300	0.1^h^	400	40	3
Rectal	300	0.1^h^	400	40	3
**Tramadol/paracetamol**					
Oral	300	0.1^h^	400	40	3
**Tapentadol**					
Oral	400	0.4	300	120	-

*Abbr.:* *DDD* Defined Daily Dose, *mg* Milligram, *oMME* oral Morphine Milligram Equivalent

*References:* ^a^ [24], ^b^ [20], ^c^ [25], ^d^ [26], ^e^ [27], ^f^ [28], ^g^ [29]

^h^ Conversion factor of tramadol was assumed to be constant across RoA

^i^ MDD of each opioid was assumed to be constant across RoA; MDDs for tilidine/naloxone, tramadol, tramadol/paracetamol were validated using package inserts

^j^ Correction factor = 120 ÷* col.*(4); extrapolates the dose assuming an MDD of 120 oMME

For each prescription, the DDD were converted to milligram and then to oMME by multiplication with conversion factors that depended on the specific OA and its RoA (see col. (1) and (2) in Table 2).

Since MDDs varied with the prescribed OA (e.g., 120 oMME/day for morphine and 60 oMME/day for tilidine/naloxone, see Col. (4)), doses of OAs with MDDs < 120 oMME were additionally multiplied by the correction factors from Table 2 (see Col. (5)). The correction factor is calculated by dividing 120 by the MDD of the specific OA. By using the correction factors, doses were standardized to an MDD of 120 oMME. This adjustment allowed for comparability between the MDD in different OA agents.

Afterwards, doses were summed over the patient's total follow-up period to obtain the total prescription dose for each patient. The prescribed daily dose for each patient was calculated by dividing the total prescription dose by the number of treatment days. The number of treatment days was defined as the timespan until the very last prescription plus each patient's median timespan between two dispensations to impute the period after the last prescription. To account for interrupted therapies, quarters without indicators of opioid intake were excluded from the number of treatment days. Quarters were assumed to show no indicators of opioid intake if no prescription was issued within that quarter and the previous quarter.

For each patient, the number of prescribed MDDs was determined by dividing the prescribed daily dose by an MDD of 120 oMME. Results > 1 indicated that the guideline-recommended MDD was exceeded on average during the observation period. For instance, a result of 2 would indicate a prescription of two MDDs, whereas a result of 0.5 would indicate a prescription of half an MDD on average. A detailed description of the stepwise calculation process can be found in Additional File 1. For comparison with existing literature, prescribed daily doses were also calculated without the correction factor.

Additionally, this calculation was repeated for 91-day intervals (follow-up quarters) to surveil doses over time. The number of prescribed MDDs within each follow-up quarter was calculated analogously to the two-year observation period including only quarters with indicators of opioid intake and assuming a duration of 91 days per quarter. A detailed description is provided in an additional file (see Additional File 1).

### Statistical analysis and covariates

Logistic regression with binary and categorical data was performed to identify predictors of exceeding the guideline-recommended MDD. Prescription characteristics were accommodated in the model using the length of prescription history, the prescription of strong opioids, benzodiazepine comedication, prescriptions by pain therapists, intoxication and the number of prescribing physicians. Alongside the prescription characteristics, age, sex and the region of residence were included in the model to analyze patient demographics (for full variable description see Additional File 2). Exceeding the guideline-recommended MDD (prescribed MDDs > 1) vs. not exceeding the guideline-recommended MDD (prescribed MDDs ≤ 1) served as the dependent variable. Predictors were assumed to be statistically significant at *p* < 0.05 and are presented as odds ratios (OR) with 95% confidence intervals (95% CI). P-values were calculated using Wald test. The regression model was tested for multicollinearity using variance inflation factors and correlation matrices. Statistical analysis was performed using Stata version 17.0.

#### Patient characteristics

For statistical analysis, various patient and prescription characteristics were defined. To acknowledge regional differences, patients were divided into four categories according to their home state. The regions contained the following categories: East (Berlin, Brandenburg, Saxony, Saxony-Anhalt, Thuringia), North (Bremen, Hamburg, Mecklenburg-Vorpommern, Lower Saxony, Schleswig–Holstein), West (Hesse, North Rhine-Westphalia, Rhineland-Palatinate, Saarland) and South (Baden-Württemberg, Bavaria).

For the logistic regression model, individuals were categorized into four age groups. Individuals aged 70 to 89 years were grouped into one age group and used as a reference category for the regression model, as this age group represents the typical age of opioid patients, accounting for more than half of the study population. The remaining age groups comprised individuals aged 18 to 49 years, 50 to 69 years, and 90 years and older.

#### Prescription characteristics

A predominantly prescribed opioid was defined as an opioid that occurred most frequently among a patient's prescriptions. For instance, if fentanyl was prescribed more frequently than other agents, fentanyl was assumed to be the patient's predominant opioid. The predominant prescription of morphine, hydromorphone, oxycodone, oxycodone/naloxone, fentanyl, buprenorphine and tapentadol were summarized as predominant prescription of strong opioids in an explanatory variable for logistic regression. Tilidine/naloxone, tramadol and tramadol/paracetamol were considered weak opioids. In Germany, these weak opioids are restricted less and are easier to access for patients and physicians [30, 31].

The length of OA prescription history was considered as variable with two categories. Individuals were classified as patients with a long-term history of OA prescriptions if they were included in the first quarter of 2018 and received opioids for four consecutive quarters in 2017. Accordingly, these patients had a history of at least 12 months of opioid therapy prior to their inclusion. Patients with fewer than four or no prescription quarters in 2017 were considered to have a short-term history.

Outpatient prescriptions of benzodiazepine in a quarter with opioid prescription were identified with the ATC-codes N05BA* or N05CD* to analyze comedication.

Physicians with additional training in pain management were identified by SHI doctor's fee schedule codes EBM 30700 and 30702. A variable for the participation of a pain therapist indicated whether at least one opioid prescription came from a physician with such additional training during the observation period.

Inpatient stays due to intoxication by opioids, other narcotics or psychodysleptics were identified by ICD-10-GM T40.X (poisoning by narcotics and psychodysleptics, including opioids, synthetic narcotics, unspecified narcotics, cannabis, lysergide (LSD), and unspecified psychodysleptics) or F11.0 (acute opioid intoxication).

### Ethical approval and registration

The study was approved by the ethics committee of the medical faculty of the University of Duisburg-Essen (ref. no. 21–9964-BO) and included in the German Clinical Trials Register (ID DRKS00024854). The German Federal Office for Social Security consented the use of social insurance data.

## Results

A total of 1,756,599 prescriptions were issued over the course of the observation period. These prescriptions were received by 113,475 patients, representing approximately 2% of all persons insured by *DAK-Gesundheit* in 2018 [21]. The mean number of prescriptions received by patients was 15.5 (standard deviation (SD) = 11.3; max. = 744), with an average of 1.1 RoA per patient (SD = 0.4; max. = 5). This suggests that for the majority of patients, only one RoA was utilized. The average age was 71.8 years with an SD of 14.4 (18–49 years: *n* = 8,571 (7.5%); 50–69 years: *n* = 35,734 (31.5%); 70–89 years: 60,136 (53.0%); ≥ 90 years: *n* = 9,034 (8.0%)). The percentage of females was 74.6%.

The mean number of prescribing physicians per patient was 2.3 (SD = 1.5; max. = 58). Approximately 49.1% (*n* = 55,683) of the patients met the criteria for a long-term history of OA prescriptions. Approximately 12.9% of the patients received at least one benzodiazepine prescription in a quarter of the OA prescription, 15.0% received at least one prescription from a pain therapist, 45.7% met the criteria for receiving predominantly strong opioids and 0.3% were hospitalized with intoxication by opioids, other narcotics or psychodysleptics.

Table 3 shows the percentage frequencies of all prescribed opioids and RoA of all prescriptions from the two-year period. In total, 83.5% of the prescriptions were for oral opioids and 15.0% transdermal opioids, whereas parenteral, rectal, sublingual or nasal OA represented < 1%, respectively. The overall predominant opioid was oral tilidine/naloxone (31.4%), followed by oral tramadol (15.5%). Other frequently prescribed opioids were transdermal fentanyl (11.5%), oral oxycodone (10.1%) and oxycodone/naloxone (8.5%).

**Table 3:** Prescribed Opioids and RoA (*n*=1,756,599 prescriptions)

	**Total**	**O**	**P**	**R**	**TD**	**SL**	**N**
Morphine	4.72%	4.26%	0.46%	<0.01%			
Hydromorphone	7.22%	7.14%	0.08%				
Oxycodone	10.10%	10.10%	0.01%				
Oxycodone/naloxone	8.49%	8.49%					
Fentanyl	11.80%				11.54%	0.22%	0.03%
Buprenorphine	4.01%		0.02%		3.44%	0.54%	
Tilidine/naloxone	31.41%	31.42%					
Tramadol	15.66%	15.52%	0.07%	0.07%			
Tramadol/paracetamol	1.05%	1.05%					
Tapentadol	5.54%	5.54%					
**Total**	100.00%	83.51%	0.64%	0.08%	14.98%	0.76%	0.03%

*Abbr.: O* Oral, *P* Parenteral, *R* Rectal, *TD* Transdermal, *SL* Sublingual, *N* Nasal, *RoA* Route of Administration

### Prescribed doses and exceeding the guideline-recommended MDD

About 10.5% of the patients exceeded the guideline-recommended MDD on a two-year average according to their OA prescriptions. The average prescribed number of MDDs within the study population was 0.48 (SD = 0.66; median = 0.28; max. = 30.05) indicating the prescription of approximately a half MDD on average. The average prescribed daily dose was 43.6 oMME (SD = 75.8; median = 20.0; max. = 3605.5).

As shown in Table 4, most of the patients received only one opioid agent within the follow-up period, whereas 4.7% of the patients received two weak OAs, 11.7% one weak and one strong opioid, 6.6% two strong opioids and 9.7% more than two different opioids. The only opioid with an average prescribed MDD > 1 was hydromorphone (mean = 1.04; SD = 1.03). Fentanyl only (mean = 0.80; SD = 0.83) and two strong opioids (mean = 0.87; SD = 1.04) were the combinations with the next highest average prescribed MDDs. Prescriptions of weak OA led to smaller means and smaller standard deviations. In detail, patients receiving only tilidine/naloxone had an average prescribed MDD of 0.24 (SD = 0.22), those receiving only tramadol 0.43 (SD = 0.43) and those receiving only tramadol/paracetamol 0.13 (SD = 0.09).

**Table 4:** Patterns of opioid combinations and number of prescribed MDDs (*n*=113,475 patients)

		**Prescribed MDDs**		
	**%**	**Mean**	**SD**	**Median**
Morphine only^a^	2.0%	0.70	1.01	0.43
Hydromorphone only^a^	3.5%	1.04	1.03	0.73
Oxycodone only^a^	4.9%	0.71	1.02	0.37
Oxycodone/naloxone only^a^	4.4%	0.67	0.57	0.49
Fentanyl only^a^	6.4%	0.80	0.83	0.53
Buprenorphine only^a^	2.6%	0.36	0.38	0.20
Tilidine/naloxone only	27.1%	0.24	0.22	0.17
Tramadol only	12.3%	0.43	0.43	0.31
Tramadol/paracetamol only	0.7%	0.13	0.09	0.10
Tapentadol only^a^	3.2%	0.15	0.10	0.13
2 weak OA	4.7%	0.27	0.29	0.19
1 weak + 1 strong OA	11.7%	0.44	0.54	0.29
2 strong OA	6.6%	0.87	1.04	0.55
>2 OA	9.7%	0.58	0.81	0.37

^a^Strong opioid

Approximately 63.3% of the patients were treated with OAs by a general practitioner (GP) only and thereby had an average of 0.45 (SD = 0.58) prescribed MDDs. Patients with the highest average prescribed MDDs were those treated with OA by anesthesiologists only (mean = 0.64; SD = 0.78), by anesthesiologists and GP (mean = 0.69; SD = 0.88) or by more than two specialist groups (mean = 0.64; SD = 1.13). Orthopedists/trauma surgeons (mean = 0.30; SD = 0.45) and orthopedists/trauma surgeons with GP (mean = 0.35; SD = 0.45) exhibited the lowest average prescribed MDDs. Detailed results are provided in an additional file (see Additional File 3).

### Predictors of exceeding the guideline-recommended MDD

The results of the logistic regression analysis are shown in Table 5. Results were assumed statistically significant for a p-value below 0.05. We found that for the 18–49 years old the odds of exceeding the guideline-recommended MDD increased by 187% (OR = 2.87, *p* < 0.001) and for the 50–69 years old by 90% (OR = 1.90, *p* < 0.001) compared to the 70–89-years-old, whereas the odds for patients aged 90 years and older decreased by 36% (OR = 0.64, *p* < 0.001). Female sex was associated with a 27% reduction in the odds of exceeding the guideline-recommended MDD (OR = 0.73, *p* < 0.001). Among patients from the northern (OR = 1.08, *p* < 0.05) and eastern federal states (OR = 1.08, *p* < 0.05) the odds increased by 8% compared to patients from the southern part of Germany. The result for the western region was not statistically significant (OR = 0.99, *p* = 0.72). For patients with long-term history of OA prescriptions the odds of exceeding increased by more than fivefold (OR = 5.35, *p* < 0.001) and for those who received predominantly strong opioids by more than sixfold (OR = 6.79, *p* < 0.001). Comedication with benzodiazepine was associated with a 48% increase (OR = 1.48, *p* < 0.001) and prescriptions by pain therapists with a 19% increase (OR = 1.19, *p* < 0.001) in the odds of exceeding. For patients with OA intoxications related to opioids, other narcotics or psychodysleptics, the odds of exceeding the guideline-recommended MDD more than doubled (OR = 2.17, *p* < 0.001). Patients who received prescriptions from 5–7 physicians had 81% higher odds of exceeding the guideline-recommended MDD compared to patients with 1–4 physicians (OR = 1.81, *p* < 0.001). The odds increased by more than sevenfold for those who received prescriptions from > 7 physicians (OR = 7.66, *p* < 0.001).

**Table 5:** Predictors of exceeded guideline-recommended MDDs on a two-year average, calculated using logistic regression

	**Logistic regression**		**Frequencies**			
			**Exceeding (*n*=11,870)**		**Non-exceeding (*n*=101,605)**	
	**OR**	**[95% CI]**	***n***	**(%)**	***n***	**(%)**
Age						
18-49 years	2.87***	[2.66-3.09]	1,437	(12.11%)	7,134	(7.02%)
50-69 years	1.90***	[1.81-1.99]	5,041	(42.47%)	30,693	(30.21%)
70-89 years	Reference		4,845	(40.82%)	55,291	(54.42%)
≥90 years	0.64***	[0.58-0.70]	547	(4.61%)	8,487	(8.35%)
Female	0.73***	[0.69-0.76]	8,181	(68.92%)	76,423	(75.22%)
Regions						
North	1.08*	[1.01-1.15]	3,175	(26.75%)	23,343	(22.97%)
East	1.08*	[1.00-1.15]	2,132	(17.96%)	16,890	(16.62%)
South	Reference		2,226	(18.75%)	22,393	(22.04%)
West	0.99	[0.93-1.05]	4,337	(36.54%)	38,979	(38.36%)
Long-term opioid history	5.35***	[5.06-5.66]	10,132	(85.36%)	45,551	(44.83%)
Strong opioids	6.79***	[6.43-7.16]	9,926	(83.62%)	41,914	(41.25%)
Comedication with benzodiazepine	1.48***	[1.40-1.57]	2,189	(18.44%)	12,426	(12.23%)
Prescription by pain therapist	1.19***	[1.13-1.25]	2,915	(24.56%)	14,072	(13.85%)
Intoxication (ICD-10 T40.-, F11.0)	2.17***	[1.69-2.68]	124	(1.04%)	266	(0.26%)
No. of prescribing physicians						
1-4 physicians	Reference		10,272	(86.54%)	95,799	(94.29%)
5-7 physicians	1.81***	[1.68-1.95]	1,264	(10.65%)	5,427	(5.34%)
>7 physicians	7.66***	[6.40-9.16]	334	(2.81%)	379	(0.37%)
Intercept	0.00***	[0.00-0.00]				

*** *p*<.001; ** *p*<.01; *p<.05; McFadden’s pseudo R² = 0.23; *n* = 113,475; variable description see Additional File 1

### Observation of prescribed MDDs over time

**Fig. 1:**
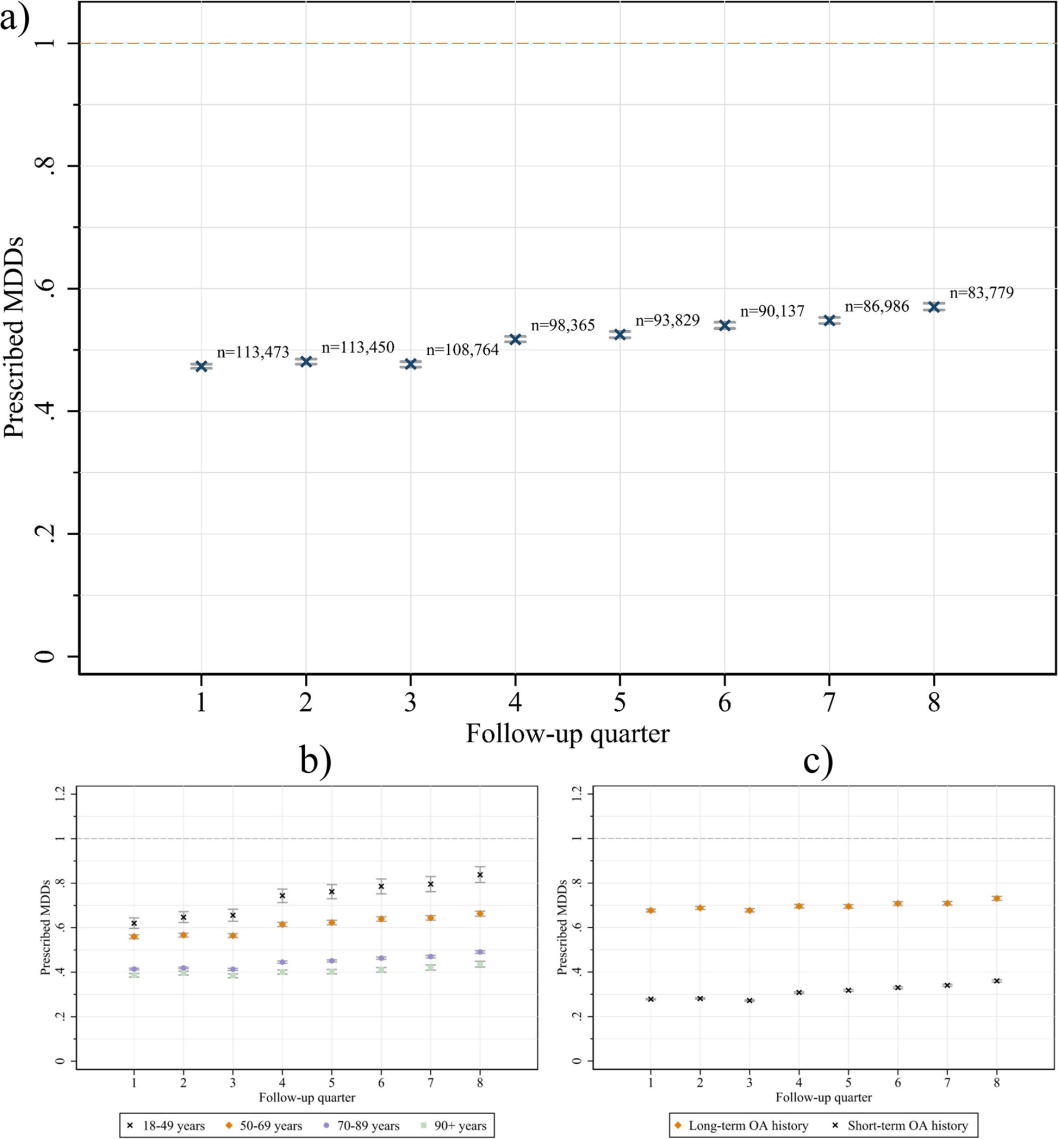
**a** Average number of prescribed MDDs with 95% CI per quarter and number of patients with indicators of opioid intake per quarter. Two patients were not included in follow-up quarter 1 because the prescribed opioid was dispensed in quarter 2. **b** Prescribed MDDs by age groups and (**c**) by OA prescription history

Figure 1a shows the average prescribed MDD with 95%-CI (y-axis) and the number of patients with indicators of opioid intake for each follow-up quarter (x-axis). As shown in the figure, the average prescribed MDD increased slightly on a quarterly basis over the course of two years. When comparing age groups, the amount of prescribed MDDs increased steeper among younger groups (see Fig. 1b). Patients with short-term OA history showed a slightly steeper increase compared to patients with long-term OA history (see Fig. 1c).

**Fig. 2:**
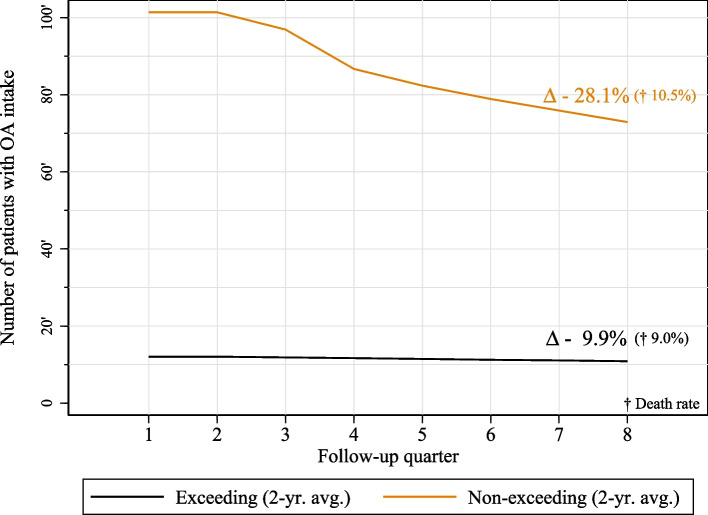
Number of patients indicative of opioid intake per quarter. Grouped by exceeding and non-exceeding patients including total decline (Δ) and death rate (†) within observation period

Figure 2 shows the number of patients with indicators of OA intake per quarter for the patients exceeding and for the patients not exceeding the guideline-recommended MDD. In the non-exceeding group, the number of patients decreased by 28.1%. The number of patients among the exceeding decreased by 9.9%, whereas the death rate was comparable for both groups (10.5% vs. 9.0%).

## Discussion

The study population consisted of 113,475 patients who received LTOT, with a majority being female (74.6%) and elderly (average age of 71.8 years). Overall, 10.5% of these patients exceeded the guideline-recommended MDD during the observation period on average. Similar results were obtained for Germany with 15.5% exceeding 100 oMME/day in 2012 [16] and 9.9% exceeding 120 oMME/day in 2014 [2] despite using different dose thresholds, definitions of LTOT, and observation periods. British studies have determined a proportion of 23.3% receiving > 100 oMME/day between 2000 and 2010, and 19.6% receiving > 90 oMME/day in 2019 [32, 33]. Whereas all the other studies we identified focused on the prescribed doses in terms of oMME, our approach aimed to address agent-related MDDs and whether these were exceeded. The average prescribed daily dose of 43.6 oMME in our findings was lower or in line compared to other research, such as 58.0 oMME/day [16] and 48.0 oMME/day in Germany [2], and around 40.0 oMME/day in the US [34].

The number of patients exceeding and the average number of prescribed MDDs did not indicate an increasing trend of high-dose prescriptions compared to past analyses [2, 16] and support the conclusions that there are still no signs of an opioid crisis in Germany [7, 8]. However, our findings show a high variation of doses and could therefore raise awareness of certain prescribing patterns and affected patient groups. Exceeding the MDD may be medically justified if a patient benefits from a higher dose. Still, also smaller doses of 50 to 100 oMME/day can increase the risk of adverse events such as OA-related overdoses [35], death [36], and road trauma [37].

The German guideline on LTOT for CNCP recommends an MDD of 120 oMME but additionally specifies smaller MDDs for some OAs in a practice tool [1, 20]. With regard to dosage, other guidelines recommend different approaches. The French guideline recommends not to exceed 150 oMME/day [38], the Canadian guideline a maximum of 90 oMME/day at the beginning of therapy [39, 41], while the US guideline states that dose increases above 50 oMME/day should be treated with caution [40]. However, all of these guidelines emphasize reasonable exceptions depending on therapeutic response.

### Factors associated with exceeding the guideline-recommended MDD

#### Patient characteristics

Younger age and male sex were found to be moderately associated with exceeding the guideline-recommended MDD, as they were for high-dosed OA therapy in other research [2, 16, 32]. Given the association between high-dose therapy and opioid-related harms [41], it raises the question of whether the recommended regular evaluation of benefits and harms [1] should be more prioritized, particularly in these groups of patients.

Individuals from the northern and eastern parts of Germany showed slightly higher odds of exceeding the guideline-recommended MDD compared to the southern federal states. This is consistent with another study that found higher prescription volumes of strong OA in northern and eastern Germany than in western and southern Germany [42]. However, our grouping of the federal states into four regions may be too imprecise to determine regional trends. As regional differences in healthcare may be important for resource allocation, future research should analyze areas at a smaller scale and identify drivers of these discrepancies (e.g., morbidity structure, material resources).

#### Patients with long-term OA history

A history of at least 12 months of opioid prescriptions prior to inclusion was strongly associated with exceeding the guideline-recommended MDD. These results are consistent with the trends shown by other research. In a study by Chevalier et al. the highest daily doses among German patients were found among those with the longest duration of opioid therapy, whereas this association could not be found among British patients [43]. The patients within our sample were all considered long-term patients (prescriptions for > 3 months), but those who had been receiving prescriptions for longer periods (e.g., > 12 months prior to inclusion) tended to have higher doses than others. In some cases, patients continue opioid therapy to avoid painful withdrawal [44, 45] or experience tolerance development, which may be accompanied by a dose increase [13, 46]. Our results reinforce the significance of certain guideline-recommended measures, such as contemplating a dosage reduction after a six-month period or reevaluating therapy in the event of diminishing effectiveness [1].

#### Number of prescribing physicians

A high number of prescribing physicians was associated with exceeding the guideline-recommended MDD – particularly for the group with > 7 physicians in two years. Another German study showed an association of more than two prescribers in one year with doses ≥ 100 oMME/day [16]. This might indicate a high demand for treatment on the one hand. On the other hand, it might reflect a need for a greater variety of options for interdisciplinary consultation and communication between physicians, which could potentially avoid unintended multiple prescriptions or doctor shopping.

#### Prescription by pain therapist and other specialist groups

Prescriptions by physicians with additional training in pain management were weakly associated with exceeding the guideline-recommended MDD. The results suggest that pain therapists may not be strongly involved in high-dose prescribing. Given international guideline recommendations, this could imply a need for an enhancement of interdisciplinary consultation in high-dose LTOT in Germany. According to the Canadian guideline, a second opinion may be appropriate for doses > 90 oMME/day [39], while the French guideline specifically recommends consultation with a pain specialist for doses > 150 oMME/day [38].

However, our comparison of multiple specialist groups shows that some medical specialties tended to prescribe higher doses. Patients with prescriptions involving anesthesiologists had a higher average prescribed MDD than those with prescriptions involving GPs or orthopedists and trauma surgeons (see Additional File 2). These differences may be due to the different age and morbidity structure of the patients of different specialist groups. In addition, most of the groups exhibited large standard deviations compared to the average prescribed MDD, so there may be a large heterogeneity regarding doses in each specialist group. These considerations are supported by claims data analyses from the US suggesting that not only patient characteristics but also prescriber preferences influence prescription volumes resulting in high and low volume prescribers [47]. Future research could concentrate on the influence of individual prescriber characteristics, such as preferences or training.

#### Comedication and intoxication

In our findings, comedication with benzodiazepine and inpatient stays due to intoxication by opioids, other narcotics or psychodysleptics were both statistically significantly associated with exceeding the guideline-recommended MDD, concurring with the results of other German studies [2, 16]. However, it should be noted that these studies additionally included diagnoses of abuse and dependence, and a number of additional substances, such as alcohol, hypnotics and sedatives. According to the German guideline on LTOT for CNCP, prescription of benzodiazepine is contraindicated for patients receiving OA therapy due to an elevated risk of substance-related hospital admissions or overdoses [1]. The occurrence of high-dose therapy in patients with high-risk prescribing patterns emphasizes the significance of the guideline recommendations for interdisciplinary treatment [1, 40] and close monitoring of patients with high-risk treatment [40] in order to maintain therapy safety. Furthermore, the recent introduction of the electronic patient record and electronic prescriptions in Germany may serve to reduce risky drug combinations in the future.

#### Strong opioids and opioid agents

The prescription of strong opioids was strongly associated with exceeding the guideline-recommended MDD, despite the assumption that the MDD for weak OA was exceeded at 40–60 oMME and that for strong OA at 80–120 oMME. In Germany, strong opioids are more difficult to be prescribed by physicians and harder to access by patients than weak opioids due to stricter regulations [30, 31]. In addition, the average prescribed MDD among the different opioid agents were clearly higher for strong opioids, particularly for hydromorphone, fentanyl and the combination of two strong opioids. According to the German drug prescription report, fentanyl had the highest prescription volume in terms of DDD among strong OA [6]. Our findings indicate the necessity for high awareness of dose equivalents and the potency of strong opioids, such as fentanyl or hydromorphone, to prevent unintended high-dose therapy.

### Quarterly analysis

Figure 1a showed a slight increase in the average prescribed MDD in the two-year observation period and a decline in numbers of patients with indicators of OA treatment. This decline in OA patients was mainly found among the non-exceeding patients (see Fig. 2). Accordingly, the observed increase in the average prescribed doses could be due to the discontinuation of therapy in low-dose patients, suggesting that the prescribed doses remained stable in high-dose patients who continued therapy. These results indicate that there may be at least two distinct LTOT-groups according to dose and duration of therapy. Whereas in the one group with lower doses, LTOT was discontinued more frequently, the other group was characterized by permanent high-dose opioid therapy which appeared to be mostly continued until death or the end of the observation period. It could be assumed that an uncertain proportion of the low-dose group increase in dose over time – possibly due to tolerance development [13] or pain progression – and becomes part of the high-dose group. As the increase was particularly found among the younger age groups, this may affect younger patients in particular (see Fig. 1b). Our conclusions are further supported by the slight increase in average doses among patients who started therapy more recently (short-term OA history, see Fig. 1c). However, differences between long-term (> 3 months) and permanent OA patients (≥ 2 years) could also be attributed to medical indications or therapeutic response. Close monitoring of patients on continuing high-dose therapy could be adopted from the CDC guideline on opioid prescribing [40] to assess whether patients are benefiting from high-dose therapy. Further research should examine the long-term dose increases and continuous high-dose prescribing.

### Limitations

Analyses built on SHI claims data entail several limitations. First, claims data are generated for billing purposes and thus may contain misclassification of patients due to miscoding or upcoding caused by misdiagnoses or economic incentives [48]. Furthermore, specific parameters usually collected in clinical trials (e.g. actual intake of medication, therapeutic response, underlying diseases) may not be observed [49]. The external validity of the results might be limited by differences in age, gender, and social structure between SHI funds. Nevertheless, our data cover the statutory standard care services available to all SHI enrollees.

The observation period partially overlaps with the events of the COVID-19 pandemic starting in March 2020. This might have had some impact on our results. However, the observation period of the patients included in the first quarter of 2018 (*n* = 81,315) had already ended by that time.

The authors of comparable studies may choose other dose limits, definitions of LTOT and morphine equivalent conversion factors, which could lead to divergent outcomes. For instance, the conversion factor for hydromorphone differs across various sources ranging from 4 [27] or 5 [25, 50, 51] to 7.5 [20]. The conversion factor of 7.5 used in this study indicated that hydromorphone was the opioid with the highest average prescribed MDD. A conversion factor of 5 used in a sensitivity analysis changed the average prescribed MDD from 0.48 to 0.46 and that of patients with only hydromorphone prescriptions from 1.04 to 0.70. All predictors remained steady.

The variable for hospitalizations with intoxication by opioids, other narcotics, and psychodysleptics (ICD-10 F11.0, T40.-) used in the logistic regression model also encompasses illicit substance use. In addition to opioids, ICD-10 T40.- also includes intoxications by non-opioid substances (T40.5: cocaine; T40.6: other narcotics; T40.7: cannabinoids; T40.8: LSD; T40.9: other psychodysleptics). As these non-opioid substances are also covered by other ICD-10 codes (F12.0: cannabinoids, F14.0: cocaine, F16.0: hallucinogens, F19.0: multiple substance use), the regression result (OR = 2.17) does not necessarily account for all individuals diagnosed with intoxication by these substances. It should be noted, however, that all individuals with an inpatient diagnosis of opioid-related intoxication have been included (ICD-10 F11.0, T40.1-T40.4). Consequently, the result may be shifted towards opioid-related intoxication.

The quarterly analysis and the calculation of the number of treatment-days might have led to an underestimation or overestimation of prescribed MDDs in some quarters.

## Conclusions

Our study provided an overview on the extent of exceeded guideline-recommended MDDs in LTOT among the insureds of a large German SHI company. Despite limited comparability, the scale of high-dose LTOT was similar to past German studies. In addition, no substantial alterations were observed throughout the course of treatment. Based on these results, we conclude that the MDDs of opioids are not exceeded to a problematic extent.

Still, in some patient groups high-dose LTOT is widespread, including patients with a high number of prescribing physicians or those receiving predominantly strong opioids. In particular, therapy duration and prescription history divided the study population into an exceeding and a non-exceeding group. Our results emphasize the necessity for high awareness of certain guideline recommendations, patient groups, and prescribing patterns to maintain therapy safety. However, some of the underlying circumstances, such as therapeutic response, the lack of therapeutic options, or tolerance development, remained unknown and should therefore be examined carefully.

## Supplementary Information

Additional File 1. PDF-document, .pdf. Additional File 1 – Dose calculation. Description of opioid dose calculation for the entire observation period and within follow-up quarters.

Additional File 2. PDF-document, .pdf. Additional File 2 – Variable description. Description of variables used in logistic regression.

Additional File 3. PDF-document, .pdf. Additional File 3 – Opioid prescribing patterns among specialist groups. Patterns of prescribing physicians, proportion of care provision and amount of prescribed MDDs.

## Data Availability

The usage of social security data in this study was approved by the German Federal Office for Social Security. Due to data privacy regulation, social security data cannot be made available. The data containing drug-specific information is available in the *Arzneimittel-Stammdatei plus* of the SHI drug index. Access can be requested via https://www.wido.de/publikationen-produkte/analytik/arzneimittel-klassifikation/arzneimittel-stammdatei/?L=0. [23].

## Abbreviations

CI: Confidence interval
DDD: Defined daily dose
EBM: Uniform Assessment Standard
GP: General practitioner
ICD-10-GM: International Statistical Classification of Diseases and Related Health Problems, 10th revision, German Modification
LTOT: Long-term opioid therapy
MDD: Maximum daily dose
CNCP: Chronic non-cancer pain
OA: Opioid analgesics
oMME: Oral morphine milligram equivalent
OPS: German procedural classification
OR: Odds ratio
RoA: Route of administration
SHI: Statutory health insurance
