# Supplementary material for: Exceeding the guideline-recommended maximum daily dose of opioids for long-term treatment of non-cancer pain in Germany – a large retrospective observational study
Source: BMC Public Health. 2024 Sep 27;24:2580. doi: 10.1186/s12889-024-20141-4 (PMC11429179; doi:10.1186/s12889-024-20141-4)
Supplement: Supplementary file 1 — Additional File 1. PDF-document, .pdf. Additional File 1 – Dose calculation. Description of opioid dose calculation for the entire observation period and within follow-up quarters. [file 12889_2024_20141_MOESM1_ESM.pdf]

Additional File 1: Dose calculation

Entire observation period: To determine if a patient exceeded the maximum daily dose (MDD) on average over the observation period, we used the quotient of the prescribed daily dose and the MDD. The prescribed daily dose for each patient was calculated based on Table 2 (see Methods section) and the number of treatment days. The calculation process is shown in **Figure S1.1** and explained below:

1) For each patient, the doses in DDD per package were converted to milligrams per package using the official version of the ATC classification with defined daily doses (see Col. (1) in Tab. 2).
2) The doses in milligrams per package were converted to morphine equivalents (oMME/pack) using opioid-specific conversion factors (see Col. (2) in Tab. 2).
3) Different opioids have varying MDDs. To ensure comparability between them, opioids with maximum doses less than 120 oMME/day were multiplied by a correction factor. The correction factor is calculated from the quotient of 120 oMME and the MDD of the respective opioid. Thus, the prescribed dose is extrapolated assuming an MDD of 120 oMME and made comparable across all opioids.
4) The converted and adjusted doses were summed for each patient to obtain a total prescription dose for the respective observation period.
5) The prescribed daily dose was determined by dividing the total prescription dose by the number of treatment days. The number of treatment days was based on the timespan until the last prescription and the median duration between two dispensings. Additionally, quarters without any prescriptions in the current or previous quarter were excluded from this calculation. The number of prescribed MDDs was quantified as the ratio of the prescribed daily dose to an assumed MDD of 120 oMME.

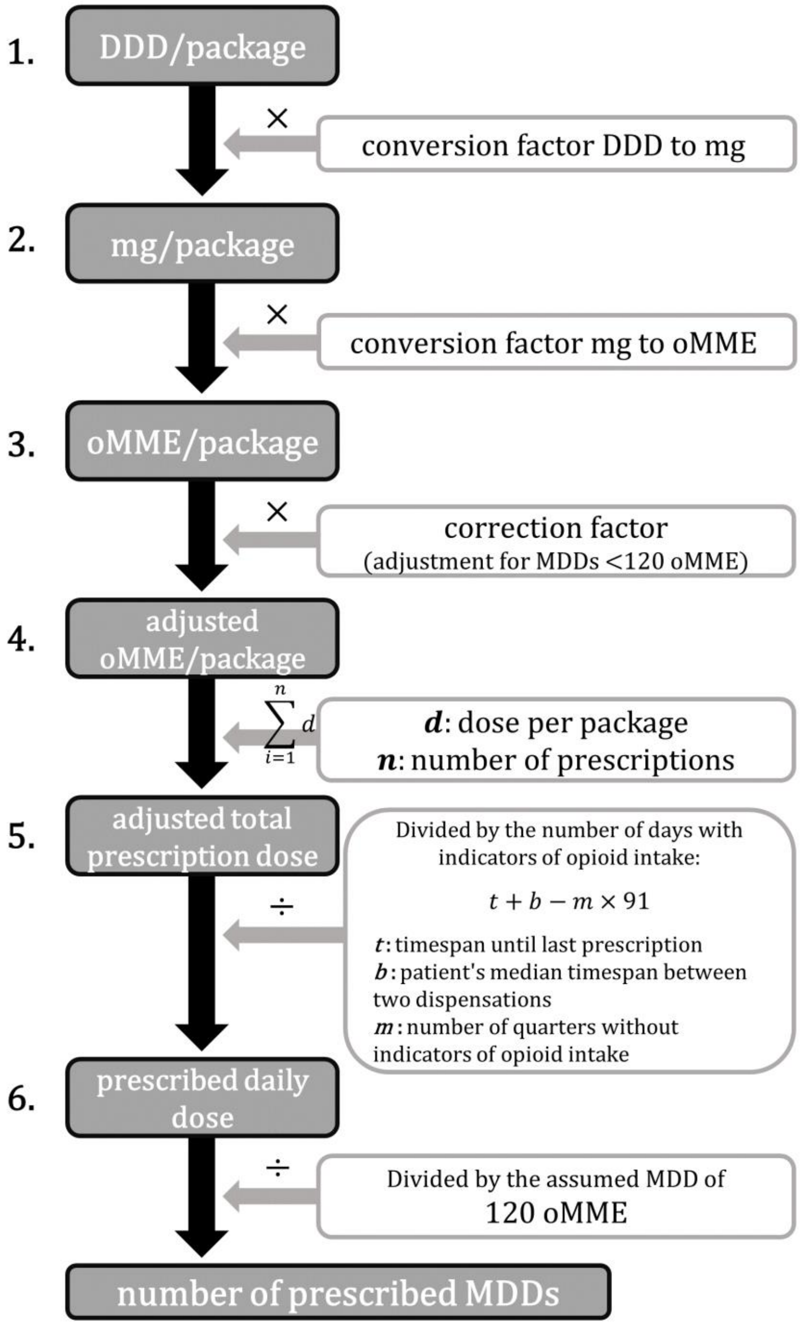

Figure S1.1: Stepwise dose conversion and calculation of prescribed MDDs. Calculations were conducted for each patient.

<u>Within follow-up quarters:</u> Measurement of the prescribed MDDs was also performed for 3-month intervals (follow-up quarters). The number of prescribed MDDs within each follow-up quarter was calculated analogously to the two-year observation period (see above) assuming a duration of 91 days per quarter (93 days for the last quarter). To avoid over- or underestimating quarterly doses, the doses of the last prescription within each follow-up quarter were attributed to quarters based on the following algorithm, which is exemplified in **Figure S1.2**:

1) The dose of the last prescription in each quarter was proportionally assigned to (i) the current quarter (e.g., quarter 1) based on the timespan until the beginning of the following quarter
2) and (ii) to the following quarter (e.g., quarter 2) based on the timespan from the beginning of that quarter until the next dispensing.
3) The combined timespans from (i) and (ii) were capped to a maximum of 91 days to account for interrupted therapies.
4) Individuals were assumed to show indicators of opioid intake in a given quarter if they received prescriptions or had doses assigned to that quarter.

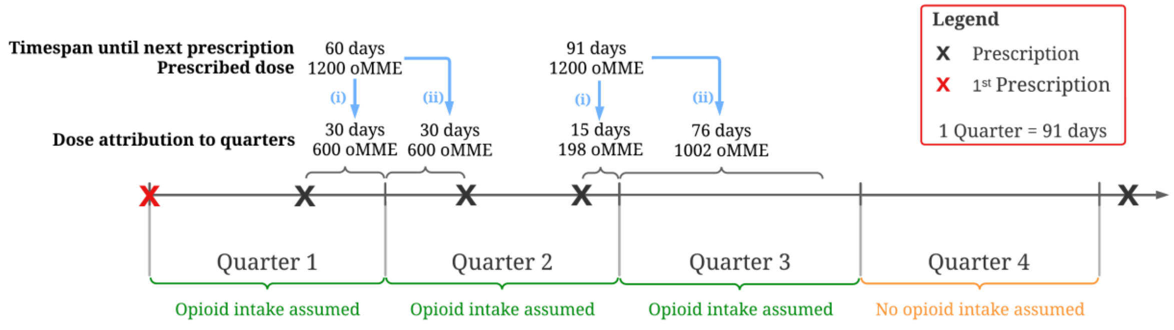

Figure S1.2: Exemplary illustration of dose calculation at the end of follow-up quarters for a single patient.
