# Supplementary material for: Exceeding the guideline-recommended maximum daily dose of opioids for long-term treatment of non-cancer pain in Germany – a large retrospective observational study
Source: BMC Public Health. 2024 Sep 27;24:2580. doi: 10.1186/s12889-024-20141-4 (PMC11429179; doi:10.1186/s12889-024-20141-4)
Supplement: Supplementary file 2 — Additional File 2. PDF-document, .pdf. Additional File 2 – Variable description. Description of variables used in logistic regression. [file 12889_2024_20141_MOESM2_ESM.pdf]

Additional File 2: Variable description

Variable	Description	Values
**Exceeded MDD***	Opioid MDD was exceeded on a two-year average (factor of exceedance >1) according to prescriptions	0 = no (Prescribed MDD ≤1)<br>1 = yes (Prescribed MDD >1)
**Age**	Age by the time of inclusion	1 = 18-49 years<br>2 = 50-69 years<br>3 = 70-89 years**<br>4 = ≥90 years
**Female**	Female sex according to self-disclosure towards SHI provider	0 = male**<br>1 = female
**Regions**	Residential state of patient by the time of inclusion, categorized into North (Bremen, Hamburg, Mecklenburg-Vorpommern, Lower Saxony, Schleswig-Holstein), East (Berlin, Brandenburg, Saxony, Saxony-Anhalt, Thuringia), West (Hesse, North Rhine-Westphalia, Rhineland-Palatinate, Saarland) and South (Bavaria, Baden-Württemberg)	1 = North<br>2 = East<br>3 = South**<br>4 = West
**Long-term opioid history**	Four quarters of opioid prescription in 2017 if patient has been included in Jan 2018 to Mar 2018	0 = no**<br>1 = yes
**Strong opioids**	Patient's most frequently prescribed opioid in the two-year period was a strong opioid (morphine, hydromorphone, oxycodone, oxycodone/naloxone, fentanyl, buprenorphine, tapentadol)	0 = weak OA**<br>1 = strong OA
**Comedication with benzodiazepine**	At least one prescription of benzodiazepine (ATC N05BA* or N05CD*) in a quarter with OA prescription	0 = no**<br>1 = yes
**Prescription by pain therapist**	At least one prescription by a physician who was licensed to bill for special pain management procedures (doctor's fee schedule EBM 30702 or 30700)	0 = no**<br>1 = yes
**Intoxication**	At least one inpatient stay due to acute opioid intoxication (ICD-10 F11.0) or narcotic substance intoxication, including opioids, synthetic narcotics, unspecified narcotics, cannabis, lysergide (LSD), and unspecified psychodysleptics (ICD-10 T40.-)	0 = no**<br>1 = yes
**No. of prescribing physicians**	Number of opioid prescribing physicians during the two-year period	1 = 1-4 physicians**<br>2 = 5-7 physicians<br>3 = >7 physicians

*Dependent variable; **reference category
