# Supplementary material for: Exceeding the guideline-recommended maximum daily dose of opioids for long-term treatment of non-cancer pain in Germany – a large retrospective observational study
Source: BMC Public Health. 2024 Sep 27;24:2580. doi: 10.1186/s12889-024-20141-4 (PMC11429179; doi:10.1186/s12889-024-20141-4)
Supplement: Supplementary file 3 — Additional File 3. PDF-document, .pdf. Additional File 3 – Opioid prescribing patterns among specialist groups. Patterns of prescribing physicians, proportion of care provision and amount of prescribed MDDs. [file 12889_2024_20141_MOESM3_ESM.pdf]

Additional File 3: Opioid prescribing patterns among specialist groups

		Prescribed MDDs		
	%	Mean	SD	Median
GP only	63.3%	0.45	(0.58)	0.28
Anesthesiologist only	3.0%	0.64	(0.78)	0.39
Orthopedist / trauma surgeon only	1.8%	0.30	(0.45)	0.16
Other specialist groups only	2.1%	0.45	(0.69)	0.25
GP & Anesthesiologist	4.6%	0.69	(0.88)	0.42
GP & orthopedist / trauma surgeon	7.4%	0.35	(0.45)	0.21
GP & other specialist groups	10.6%	0.53	(0.72)	0.32
Other combinations (containing two specialist groups)	1.5%	0.52	(0.79)	0.25
>2 specialist groups	5.7%	0.64	(1.13)	0.34
